# Machine Learning‐Based Model for Predicting Short‐ and Long‐Term Growth in Untreated Class III Malocclusion

**DOI:** 10.1111/ocr.70142

**Published:** 2026-05-18

**Authors:** Maria Denisa Statie, Michele Nieri, Valentina Rutili, Lorenzo Buffoni, Lorenzo Chicchi, Pietro Auconi, Veronica Giuntini, James A. McNamara, Lorenzo Franchi

**Affiliations:** ^1^ Department of Experimental and Clinical Medicine Università degli Studi di Firenze Firenze Italy; ^2^ Department of Physics and Astronomy Università degli Studi di Firenze Firenze Italy; ^3^ Private Practice of Orthodontics Roma Italy; ^4^ Department of Orthodontics and Pediatric Dentistry The University of Michigan Ann Arbor Michigan USA

## Abstract

**Objective:**

To develop a Machine Learning‐based model to predict growth of Caucasian subjects with untreated Class III malocclusion in the short‐ and long‐term.

**Materials and Methods:**

A longitudinal sample of 144 Caucasian subjects with untreated Class III malocclusion was selected (80% training data; 20% test data). Cephalograms of the subjects of the test group were divided into short‐ and long‐term observations. Sixteen cephalometric landmarks were digitised in an X‐Y Cartesian coordinate system. The trained model was a Graph Neural Network. A one‐sample *t*‐test and the Euclidean distances between predicted and observed values were calculated.

**Results:**

In the short‐term prediction, 16 subjects were examined. On the X‐Y axis, the following cephalometric points were statistically significant: SX, PgY, BY, PNSY, NY and SY. Mean Euclidean distance between predicted and actual values revealed high values for the mandibular points Go (2.6 mm), Me (1.9 mm), Gn (1.9 mm), Pg (2.0 mm), and B (2.0 mm). In the long‐term prediction, 13 subjects were examined. On the X‐Y axis, the following cephalometric points were statistically significant: MeX, GnX, PgX, BX, BY and PtY. Mean Euclidean distance between predicted and actual values revealed high values for the mandibular points Go (3.1 mm), Me (4.3 mm), Gn (4.1 mm), Pg (4.5 mm), and B Point (4.1 mm).

**Conclusions:**

The ML‐based prediction model was accurate for the majority of the landmarks. Cephalometric mandibular landmarks (Go, Me, Gn, Pg, and B Points) showed the highest mean Euclidean distances between predicted and observed values, indicating lower prediction accuracy.

## 1 Introduction

Growth prediction in subjects with untreated Class III malocclusion represents one of the most significant challenges in orthodontics. Over time, authors have attempted to predict the progression of mandibular growth in Class III subjects. Identifying variables as consistent predictors, however, is extremely difficult due to the complex interactions between genetic and environmental factors [1]. In addition, Class III malocclusion is not susceptible to spontaneous improvement during growth, and it tends to worsen over time, especially in males [2, 3, 4]. For these reasons, the growth pattern of Class III malocclusion is complex to assess and predict.

In the past decade, Artificial Intelligence (AI) has been integrated increasingly into dentistry, assisting clinicians in diagnosis and treatment decision‐making [5]. More recently, Machine Learning (ML), a subset of AI, along with Deep Learning (DL), has seen growing application in orthodontics [6]. ML employs algorithmic models to analyse large datasets, identifying hidden patterns or trends that can be used for predictive purposes. In contrast, DL, a specialised branch of ML, utilises artificial neural networks with multiple processing layers to extract progressively higher‐level features from data [7, 8].

Within this evolving landscape, researchers have explored AI‐driven approaches to develop algorithms and computational models for predicting mandibular growth and treatment outcomes in Class III subjects [9, 10, 11, 12, 13]. However, current findings remain incomplete, and further long‐term studies are necessary to refine these predictive models.

In medical imaging and analysis, dataset complexity is influenced by multiple factors, including the size and shape of anatomical structures, as well as age, gender, type of malocclusion, ethnic background, and bone density [14, 15, 16]. This complexity is particularly pronounced in Class III malocclusion, where these characteristics create significant variations that AI systems must recognise and interpret accurately. As a result, the increasing complexity of the dataset similarly heightens the challenge for ML models to learn effectively and generate accurate predictions [17, 18, 19].

The ability to predict a subject's growth over time is essential for accurate diagnosis and effective treatment planning. However, the existing literature highlights a significant lack of studies utilising ML to analyse growth patterns, particularly in untreated Class III malocclusion. Therefore, this study aims to develop an ML‐based model to predict growth in Caucasian subjects with untreated Class III malocclusion, considering both short‐ and long‐term evaluations.

## 2 Materials and Methods

### 2.1 Study Design

This is a retrospective, semi‐longitudinal, long‐term study investigating subjects with Class III malocclusion who did not undergo orthodontic treatment.

This study was exempted from review by the Medical School Institutional Review Board of the University of Michigan (HUM00160285).

### 2.2 Setting and Participants

This study is structured in accordance with the STROBE guidelines for longitudinal studies [20].

The present article included subjects from a previous study on craniofacial growth in untreated Class III subjects [4].

The following inclusion criteria of subjects were: Caucasian origin (European or American), no orthodontic treatment before the first and the last observation, initial diagnosis of Class III malocclusion (anterior crossbite, except pseudo‐crossbite; edge‐to‐edge incisive relationship, concomitant with one of the skeletal Class III criteria; accentuated mesial step relationship of the deciduous second molars; mesial Class III relationship of the first permanent molar of at least half cusp). A Class III skeletal relationship having one or both: A negative Wits appraisal greater than −2.0 mm; an ANB angle less than 0°. Subjects were excluded from the study if they had congenitally missing or extracted teeth or if they had craniofacial syndromes.

Each subject included had to present a series of longitudinal lateral cephalograms.

### 2.3 Data Collection and Variables

For each subject, at least two longitudinal cephalograms were available, taken at two different ages with a minimum interval of one and a half years between them. The magnification of the cephalograms ranged from 0% (life size) to 12.9% and was then standardised to 0% (life size).

Lateral cephalograms were hand‐traced during one sitting by two investigators (A.S. Levin and A.E. Zionic Alexander) in two previous articles [21, 22] and then verified for anatomic contour, landmark identification, and tracing superimpositions by a second investigator (J.A.M). Method error for cephalometric measurements was reported in Levin et al. [21] and Zionic Alexander et al. [22]. In brief, the error of the method was on average 0.6° for angular measures and 0.9 mm for linear measures [22], and intraclass correlation coefficients were extremely high [21].

A total of 16 cephalometric points Figure S1 and Table S1 were included in the study and digitised in an X‐Y Cartesian coordinate system. The cephalometric landmarks were the following: Basion (Ba), Sella (S), Nasion (N), Pterygoid point (Pt), Orbitale (Or), Anterior Nasal Spine (ANS), Posterior Nasal Spine (PNS), Point A (A), Point B (B), Pogonion (Pg), Gnathion (Gn), Menton (Me).

Gonion (Go), Articulare (Ar), Condylion (Co), Porion (Po). The X‐axis was defined as a line passing through the Nasion (N) at a 7° upward angle relative to the SN line, while the Y‐axis was a line perpendicular to the X‐axis passing through the Sella (S) point. The centre of the Cartesian axis was SX = 0 and NY = 0. X and Y coordinates of cephalometric points were expressed in millimetres in all images.

### 2.4 Sample Size

Given the retrospective nature of the study, all 144 untreated Class III subjects who met the inclusion criteria were included in the analysis.

To calculate the sample size of the test group, the desired width of the confidence interval (CI) for the difference between the observed and predicted values was used.

The desired width for the confidence interval of the Euclidean distance between predicted and actual values for the long‐term prediction was set at 3 mm. Using a 95% confidence level and an estimated standard deviation of the residuals of 2.324, at least 10 observations from the test group are required.

In the absence of preliminary data or similar studies to estimate the standard deviation of the prognostic model's residuals, a reasonable approximation was adopted. This was based on a clinically relevant interval of 12 mm, assuming a standard deviation equal to one‐fourth of that interval and a coefficient of determination (R^2^) of 0.40. Using 20% of the entire sample for the test group, it was reasonably assumed that at least 10 of the 28 randomly selected patients would meet the characteristics for inclusion in the long‐term observation group.

### 2.5 Machine Learning Model and Validation

The sample of 144 subjects was randomly divided into a training group (80% of subjects, 116 subjects) and a test group (20% of subjects, 28 subjects) using a random number generator. The model trained was a Graph Neural Network (GNN) consisting of one graph convolution layer (https://arxiv.org/abs/1609.029) and one fully connected layer for prediction. The graph was initialised with dense connectivity, and edge weights were learned during training, enabling the model to capture important correlations between different cephalometric points. The GNN received inputs including the X‐ and Y‐coordinates of all 16 cephalometric landmarks, age, gender, and the time interval between observations. The model's output was the predicted X‐ and Y‐coordinates for the subsequent observation. To enhance statistical variability and reduce the risk of overfitting, all available semi‐longitudinal observations for each subject were utilised during training, including all potential time pair combinations (e.g., T0‐T1, T1‐T2, and T0‐T2 for a subject with three observations). Small Gaussian noise was added to the X and Y coordinates to introduce random variations. The model's predictions were an average of 10 predictions from 10 noise realisations. The training objective was to minimise the mean squared error between the model's predictions and the true values in the dataset, with an added L2‐regularisation term (weight = 10^−4^) on the model parameters. The GNN was implemented using PyTorch (https://pytorch.org/) and PyTorch Geometric (https://pytorch‐geometric.readthedocs.io/en/2.5.0/index.html) and was trained from scratch. Optimisation was performed using Adam (https://arxiv.org/abs/1412.6980), a variant of Stochastic Gradient Descent, for 100 iterations with an initial learning rate of 0.002, which was reduced by a factor of 0.8 every 10 iterations.

During training, a 5‐fold cross‐validation procedure was applied to the training dataset, resulting in five models trained on different data splits. For each fold, performance was evaluated on the corresponding validation subset using the mean prediction error. Variability across folds was summarised by reporting the mean and standard deviation of the global error (2.01 ± 0.63 mm), providing an estimate of model stability across different data partitions. At the end of the cross‐validation process, the model achieving the lowest validation error was selected and subsequently applied to the independent test set, which was not used during training or validation.

The trained model was tested on the subjects in the test group, who had no prior exposure to the model. The cephalograms of the test group were selected and divided into short‐term and long‐term observations. Short‐term observation was defined as the shortest follow‐up between two consecutive cephalograms within a 1.5‐year interval. Long‐term observation was defined as the longest follow‐up, which was more than 3 years after the first observation. Predictions for the short‐term (T1) and long‐term (T2) were made using the initial observation (T0) as the input.

### 2.6 Statistical Analysis

Descriptive statistics were reported as mean and standard deviation for quantitative variables.

A one‐sample *t*‐test was conducted to evaluate the average difference between predicted and observed values on the X‐ and Y‐axes for both short‐ and long‐term observations. This test compared the mean difference against a reference value of zero (zero displacement) for the X‐ and Y‐coordinates of the 16 cephalometric points in the test group. A positive mean difference on the X‐axis indicated that the predicted value was greater (positioned further along the X‐axis) than the observed value, while a positive mean difference on the Y‐axis indicated that the predicted value was higher on the Y‐axis. Differences were considered statistically significant when *p* < 0.05. Mean absolute errors and 95% confidence intervals were also calculated for both short and long observations. Additionally, Euclidean distances between predicted and observed values were calculated for the same test subjects.

To evaluate the influence of age range and prediction length on long‐term outcomes, an analysis was conducted to correlate the age at first observation or the time interval with the Euclidean distances between predicted and observed values at points Go, Me, Gn, Pg, and B.

## 3 Results

A total of 144 subjects (65 males and 79 females, aged 2 years and 9 months up to 21 years and 7 months) were included. For each subject, a cephalogram at first observation (mean age 10.0 ± 3.7 years) and a cephalogram at last observation (mean age 13.8 years ±2.7 years) were available. Table S2 shows the demographic and cephalometric data at the first and last observations.

The sample was randomly divided into a training group of 116 subjects and a testing group of 28 subjects.

### 3.1 Short‐Term Prediction (T0‐T1)

In the short‐term prediction (T0‐T1), 16 subjects (10 females, 6 males) were included (short‐term prediction group). These subjects were selected from the test group based on having a time difference of less than 1.5 years between two observations. The other 12 subjects of the test group did not meet the criteria for the short‐term observation group because their time interval between observations was not within 1.5 years. The mean age of short‐term prediction subjects at T0 was 13.4 ± 2.8 years (range: 6.6–17.1 years), with an average time interval of 1.1 ± 0.3 years (range: 0.5–1.5 years) between T0 and T1.

The analysis of X‐axis values (Table 1) showed that only the cephalometric point SX exhibited a statistically significant difference (−0.2 mm; 95% CI: −0.3 to −0.1; *p* = 0.0085). In contrast, all other cephalometric points on the X‐axis (CoX, ArX, GoX, MeX, GnX, PgX, BX, AXE, ANSX, PNSX, NX, PoX, OrX, BaX, and PtX) did not reach statistical significance.

**TABLE 1:** Error (mm) on the X‐axis for the short‐term prediction.

	Mean	SD	95% CI Lower	95% CI Upper	*p*
CoX	0.2	0.9	−0.3	0.7	0.4489
ArX	0.1	0.9	−0.4	0.6	0.5038
GoX	−0.2	1.8	−1.1	0.8	0.7206
MeX	−0.6	1.7	−1.5	0.3	0.1476
GnX	−0.5	1.7	−1.5	0.4	0.2445
PgX	−0.5	1.7	−1.4	0.4	0.2871
BX	−0.4	1.5	−1.2	0.4	0.2865
AXE	−0.3	1.0	−0.9	0.2	0.2044
ANSX	−0.3	1.0	−0.8	0.3	0.3109
PNSX	−0.4	1.0	−0.9	0.1	0.1233
NX	0.2	0.6	−0.1	0.5	0.1509
SX	−0.2	0.2	−0.3	−0.1	0.0085*
PoX	−0.3	2.5	−1.6	1.1	0.6836
OrX	0.2	0.8	−0.2	0.7	0.2739
BaX	0.2	0.6	−0.1	0.5	0.2781
PtX	−0.1	0.9	−0.6	0.3	0.5573

Abbreviations: CI, confidence interval; SD, standard deviation.

^*^ *p* < 0.05.

For Y‐axis values (Table 2), the following cephalometric points showed statistically significant differences:

**TABLE 2:** Error (mm) on the Y‐axis for the short‐term prediction.

	Mean	SD	95% CI Lower	95% CI Upper	*p*
CoY	−0.3	1.0	−0.8	0.2	0.2419
ArY	−0.2	0.8	−0.7	0.2	0.3116
GoY	−1.1	2.2	−2.3	0.0	0.0569
MeY	−0.6	1.3	−1.3	0.1	0.0984
GnY	−0.6	1.1	−1.2	0.0	0.0559
PgY	−0.9	1.4	−1.6	−0.2	0.0187*
BY	−1.1	1.5	−1.9	−0.3	0.0114*
AY	0.1	1.4	−0.7	0.8	0.8806
ANSY	−0.0	1.3	−0.7	0.7	0.9908
PNSY	−0.7	1.3	−1.4	−0.0	0.0497*
NY	0.3	0.4	0.0	0.5	0.0274*
SY	0.7	0.7	0.3	1.0	0.0012*
PoY	−0.1	0.5	−0.3	0.2	0.6479
OrY	−0.3	0.6	−0.7	0.1	0.1023
BaY	−0.3	0.6	−0.6	0.0	0.0704
PtY	−0.1	0.8	−0.5	0.3	0.6597

Abbreviations: CI, confidence interval; SD, standard deviation.

^*^ *p* < 0.05.

PgY (−0.9 mm; 95% CI: −1.6 to −0.2; *p* = 0.0187), BY (−1.1 mm; 95% CI: −1.9 to −0.3; *p* = 0.0114), PNSY (−0.7 mm; 95% CI: −1.4 to 0.0; *p* = 0.0497), NY (0.3 mm; 95% CI: 0.0 to 0.5; *p* = 0.0274), SY (0.7 mm; 95% CI: 0.3 to 1.0; *p* = 0.0012). Conversely, the cephalometric points CoY, ArY, GoY, MeY, GnY, AY, ANSY, PoY, OrY, BaY, and PtY did not show statistically significant differences.

The mean Euclidean distance between predicted and observed values in the short‐term prediction (T0‐T1) (Table 3) indicated the highest deviations at the following mandibular points: Go (2.6 mm), Me (1.9 mm), Gn (1.9 mm), Pg (2.0 mm), B (2.0 mm). In contrast, cephalometric points with the lowest mean Euclidean distance (< 1.0 mm) included N, S, Or, Ba (Table 4).

**TABLE 3:** Euclidean distance (mm) between predicted and actual values for the short‐term prediction.

Landmark	Mean	SD	95% CI Lower	95% CI Upper
Co	1.2	0.7	0.8	1.6
Ar	1.1	0.5	0.8	1.4
Go	2.6	1.4	1.9	3.3
Me	1.9	1.3	1.2	2.5
Gn	1.9	1.1	1.3	2.5
Pg	2.0	1.2	1.4	2.7
B	2.0	1.3	1.3	2.7
A	1.4	0.9	1.0	1.9
ANS	1.3	0.9	0.9	1.8
PNS	1.7	0.7	1.3	2.0
N	0.7	0.4	0.5	0.9
S	0.8	0.5	0.5	1.1
Po	1.6	2.0	0.5	2.7
Or	0.9	0.5	0.6	1.2
Ba	0.8	0.4	0.6	1.0
Pt	1.0	0.5	0.8	1.3

Abbreviations: CI, confidence interval; SD, standard deviation.

**TABLE 4:** Error (mm) on the X‐axis for the long‐term prediction.

	Mean	SD	95% CI Lower	95% CI Upper	*p* value
CoX	0.2	1.1	−0.5	0.9	0.6207
ArX	−0.1	1.4	−1.0	0.7	0.7239
GoX	0.2	1.9	−0.9	1.3	0.6958
MeX	−2.2	3.5	−4.3	−0.1	0.0421*
GnX	−2.2	3.2	−4.1	−0.2	0.0315*
PgX	−2.3	3.2	−4.3	−0.3	0.0253*
BX	−1.9	2.2	−3.3	−0.6	0.0093*
AXE	−0.6	1.6	−1.6	0.4	0.2016
ANSX	−0.5	1.9	−1.7	0.6	0.3288
PNSX	0.4	1.0	−0.2	1.0	0.2102
NX	0.3	1.1	−0.4	1.0	0.3423
SX	−0.1	0.3	−0.2	0.1	0.5716
PoX	−0.2	1.5	−1.1	0.7	0.6159
OrX	0.3	1.7	−0.7	1.4	0.4875
BaX	−0.1	1.2	−0.9	0.6	0.7148
PtX	0.1	1.2	−0.6	0.8	0.7300

Abbreviations: CI, confidence interval; SD, standard deviation.

^*^ *p* < 0.05.

Mean absolute errors on the X‐axis and on the Y‐axis were reported in Tables S3 and S4.

### 3.2 Long‐Term Prediction (T0‐T2)

For the long‐term prediction (T0‐T2), 13 subjects (10 females, 3 males) were included (long‐term prediction group). These subjects were selected from the test group because their follow‐up from the first to the last observation was more than 3 years. The other 15 subjects from the test group did not meet the criteria for the long‐term observation group because the time interval between their first and last observations was less than 3 years. The mean age of the long‐term prediction subjects at T0 was 10.5 ± 2.2 years (range: 6.7–13.2 years), with an average time interval of 5.1 ± 2.0 years (range: 3.2–10.3 years) between T0 and T2.

The analysis of X‐axis values (Table 4) identified the following cephalometric points as statistically significant: MeX (−2.2 mm; 95% CI: −4.3 to −0.1; *p* = 0.0421), GnX (−2.2 mm; 95% CI: −4.1 to −0.2; *p* = 0.0315), PgX (−2.3 mm; 95% CI: −4.3 to −0.3; *p* = 0.0253), BX (−1.9 mm; 95% CI: −3.3 to −0.6; *p* = 0.0093).

Conversely, the cephalometric points CoX, ArX, GoX, AXE, ANSX, PNSX, NX, SX, PoX, OrX, BaX, and PtX did not reach statistical significance.

For Y‐axis values (Table 5), the cephalometric points with statistically significant differences were: BY (−2.9 mm; 95% CI: −4.7 to −1.1; *p* = 0.0047) and PtY (−0.4 mm; 95% CI: −0.8 to −0.1; *p* = 0.0229). All other cephalometric points (CoY, ArY, GoY, MeY, GnY, PgY, AY, ANSY, PNSY, NY, SY, PoY, OrY, and BaY) were not statistically significant.

**TABLE 5:** Mean error (mm) on the Y‐axis for the long‐term prediction.

	Mean	SD	95% CI Lower	95% CI Upper	*p*
CoY	0.1	1.0	−0.6	0.7	0.8158
ArY	0.2	1.0	−0.4	0.9	0.3984
GoY	0.1	3.1	−1.8	2.0	0.8950
MeY	−0.3	3.1	−2.1	1.6	0.7541
GnY	−0.8	3.0	−2.7	1.0	0.3420
PgY	−1.7	3.2	−3.6	0.3	0.0882
BY	−2.9	3.0	−4.7	−1.1	0.0047*
AY	0.0	2.4	−1.4	1.5	0.9538
ANSY	−0.3	2.3	−1.7	1.0	0.5909
PNSY	−0.2	1.1	−0.9	0.5	0.5094
NY	0.2	0.5	−0.1	0.5	0.2462
SY	0.4	1.2	−0.3	1.1	0.2427
PoY	−0.3	0.8	−0.7	0.2	0.2488
OrY	−0.2	0.8	−0.7	0.3	0.4218
BaY	−0.1	1.0	−0.8	0.5	0.6975
PtY	−0.4	0.6	−0.8	−0.1	0.0229*

Abbreviations: CI, confidence interval; SD, standard deviation.

^*^ *p* < 0.05.

The mean Euclidean distance between predicted and observed values (Table 6) revealed the greatest deviations at the following mandibular points: Go (3.1 mm), Me (4.3 mm), Gn (4.1 mm), Pg (4.5 mm).

**TABLE 6:** Mean Euclidean distance (mm) between predicted and actual values for the long‐term prediction.

Landmark	Euclidean distance	SD	95% CI Lower	95% CI Upper
Co	1.3	0.8	0.8	1.8
Ar	1.5	0.8	1.0	2.0
Go	3.1	1.8	2.0	4.1
Me	4.3	2.6	2.7	5.9
Gn	4.1	2.7	2.5	5.7
Pg	4.5	2.7	2.9	6.2
B	4.1	3.0	2.3	5.9
A	2.6	1.4	1.7	3.4
ANS	2.4	1.7	1.4	3.4
PNS	1.4	0.7	1.0	1.8
N	1.1	0.7	0.6	1.5
S	1.0	0.8	0.6	1.5
Po	1.5	0.7	1.1	2.0
Or	1.6	1.0	1.0	2.2
Ba	1.3	0.8	0.8	1.8
Pt	1.3	0.4	1.1	1.5

Abbreviations: CI, confidence interval; SD, standard deviation.

B (4.1 mm). In contrast, cephalometric points with the lowest mean Euclidean distance (≤ 1.5 mm) included Co, Ar, PNS, N, S, Po, Ba, Pt (Table 6).

Mean absolute errors on the X‐axis and on the Y‐axis were reported in Tables S5 and S6.

Pearson correlation coefficients (*r*) between the Euclidean distances (predicted vs. observed values) and both age at first observation and follow‐up duration for the landmarks Go, Me, Gn, Pg, and B are reported in Table S7. Correlations between age at first observation and Euclidean distances were consistently negative, suggesting that prediction may be more challenging in younger patients at baseline; however, none of these associations reached statistical significance (*p* > 0.142). Likewise, correlations between the follow‐up interval (time between first and last observation) and Euclidean distances were positive for all landmarks except Go (*r* = −0.07), indicating a trend toward increased prediction difficulty with longer intervals. These correlations were also not statistically significant (*p* > 0.329).

## 4 Discussion

This study aimed to develop an ML‐based model for predicting short‐ and long‐term growth in Caucasian subjects with untreated Class III malocclusion.

An accurate understanding of an individual's growth pattern is crucial for predicting future development and establishing general inductive rules [23]. This is particularly relevant in orthodontics, where accurate diagnosis and growth assessment guide treatment planning [12]. Over the years, various predictive methods have been explored for forecasting Class III growth [24], yet recent advancements in artificial intelligence (AI) have helped overcome several inherent limitations in this field.

This study employed a Graph Neural Network (GNN), a type of machine learning model designed to predict cephalometric point displacement on a Cartesian plane. GNNs capture graph dependencies via message passing between nodes, mimicking the way neurons in the human brain process information to identify patterns and reach conclusions [25]. The model was tested for both short‐term predictions (within 1.5 years from T0) and long‐term predictions (more than 3 years from T0).

In the short‐term analysis, statistical significance was observed for SX on the X‐axis and PgY, BY, PNSY, NY, and SY on the Y‐axis. However, only BY exhibited a displacement greater than 1 mm (−1.1 mm) (Tables 1 and 2).

Although several cephalometric points showed statistically significant differences between predicted and observed values, the magnitude of these differences was often small (frequently below 1 mm). This highlights the distinction between statistical significance and clinical relevance. In cephalometric analysis, such small discrepancies are generally considered to have limited clinical impact and are unlikely to influence treatment decisions when evaluated within the broader context of craniofacial growth patterns. Therefore, the interpretation of statistically significant findings in this study should be guided not only by *p*‐values but also by the magnitude and clinical relevance of the observed differences.

The mean Euclidean distance was notably higher (> 1.0 mm) for mandibular points Go, Me, Gn, Pg, and B, with Gonion (Go) showing the highest deviation (2.6 mm). In contrast, N, S, Or, and Ba exhibited the lowest mean Euclidean distances, indicating a closer alignment between predicted and observed values (Table 3).

For the long‐term prediction, four cephalometric points on the X‐axis were statistically significant, with displacements of approximately 2 mm (MeX, GnX, PgX, and BX). On the Y‐axis, BY (−2.9 mm) and PtY (−0.4 mm) were significant, with BY showing the largest difference (Tables 4 and 5).

The mean Euclidean distance in the long‐term prediction revealed even higher deviations for Go, Me, Gn, Pg, and B, with Pg showing the highest error (4.5 mm). In contrast, point S demonstrated the lowest mean Euclidean distance (< 1 mm) (Table 6).

This study highlights the limited predictability of mandibular regions, particularly for the gonial angle and for the symphyseal mandibular region in both the short term and the long term. These findings suggest that predicting mandibular cephalometric points is more challenging than predicting cranial base landmarks—especially Sella (S), which consistently exhibited lower prediction errors across both short‐ and long‐term analyses. In both short‐ and long‐term predictions, mandibular measurements were less accurate than those of other craniofacial structures. The increase in Euclidean distances over time suggests that as individuals grow, the model faces greater difficulty in accurately forecasting cephalometric changes.

Several studies have explored AI‐based mandibular growth prediction. Kim et al. [26] used AI to predict cephalometric landmark coordinates at age 13 based on data from ages 6 to 12, reporting the highest errors for Gonion, Menton, Gnathion, Pogonion, and B‐point. Similarly, Condylion, Articular point, A‐point, SNA point, and Orbital point exhibited errors ranging from 2 to 3 mm, confirming the challenges of predicting mandibular growth over time.

A study by Jiwa [9] found a considerable difficulty in predicting anatomical landmarks along the anterior border of the mandible with a Deep Learning Algorithm (DLA). In particular, the prediction of the future position of the gonion as DLA's error was particularly high (5.24 mm). This large error can be due to several factors that make manual landmark positioning of the gonion difficult: Human error in selecting its position along a gradual curve, intersecting the point if borders are not superimposed, and changes in film orientation across time points [9, 27].

Despite the limited predictability of mandibular points, Zhang et al. [28] developed an AI model to predict mandibular growth in children, using heat maps to enhance prediction accuracy. Their findings emphasised that AI primarily focuses on the chin, lower edge of the mandible, incisors, airway, and condyle—regions crucial for mandibular growth regulation [29]. However, in untreated Class III malocclusion, the complexity of growth patterns makes mandibular predictions inherently more challenging, as confirmed by the greater mean Euclidean distances in this study. Cephalometric landmark detection is an operator‐dependent procedure which is not free of interpretation by the operator, which can increase error [9].

The magnitude of prediction errors observed in this study, particularly for mandibular landmarks in the long‐term analysis (approximately 3–4.5 mm), should be interpreted in light of the biological variability and complexity of mandibular growth in untreated Class III subjects. Previous studies [9, 26, 28] have similarly reported greater prediction uncertainty in the mandibular region, especially for landmarks such as Gonion, Menton, Gnathion, and Pogonion, which are known to be more difficult to predict due to their dynamic growth patterns and sensitivity to individual variability. From a clinical perspective, these error values do not necessarily reflect a lack of usefulness of the model, but rather the intrinsic uncertainty associated with long‐term growth prediction. In orthodontic decision‐making, treatment planning is typically based on overall skeletal trends rather than the exact position of individual landmarks. Therefore, the model should be interpreted as a tool to support clinical judgment by providing an estimate of growth tendencies and associated uncertainty, rather than precise point‐to‐point predictions. Greater caution is warranted when interpreting long‐term mandibular predictions, while the model appears more reliable for cranial base structures and short‐term assessments.

Most existing AI studies on Class III malocclusion prediction have been conducted on Asian populations, while Caucasian samples remain underrepresented [30]. Understanding craniofacial growth variations across ethnic groups [31, 32] is essential for ensuring accurate predictions and generalisability. Additionally, many AI‐based studies have focused on predicting growth up to adolescence, whereas research has shown that Class III malocclusion tends to worsen over time, particularly in males, up to age 21 [4]. Expanding predictive models to include long‐term observations is crucial for improving clinical decision‐making. This study is the first to assess long‐term Class III growth prediction in a Caucasian sample, with data spanning from 18 years up to 21 years and 7 months. Unfortunately, there were only 3 males in the long‐term prediction test group, and this could be a limitation of this study. An additional limitation is that the cervical vertebrae were not traced. Therefore, it was not possible to determine the skeletal maturation stage using the cervical vertebral maturation method.

An additional limitation of the present study is the relatively small sample size of the test group, particularly for the short‐term (*n* = 16) and long‐term (*n* = 13) subgroups. While this reflects the inherent difficulty of collecting longitudinal data in untreated Class III subjects due to ethical and logistical constraints, it may affect the robustness of the model's performance estimates. In particular, smaller sample sizes may increase variability and reduce the stability of prediction errors, especially in the long‐term analysis where growth patterns are more complex and heterogeneous. Although strategies such as 5‐fold cross‐validation, data augmentation, and regularisation were implemented during model development to mitigate overfitting and enhance generalisation, the external validity of the model remains limited. Therefore, caution is warranted when extrapolating these findings to broader populations or different ethnic groups. Future studies based on larger and more diverse longitudinal datasets are needed to further validate and refine the predictive performance of the model.

Future advancements in AI and predictive algorithms may help overcome the limitations posed by small sample sizes, ultimately improving the accuracy and reliability of growth prediction models for Class III malocclusion.

## 5 Conclusions

The ML‐based prediction model was accurate for the majority of the landmarks. Cephalometric mandibular landmarks (Go, Me, Gn, Pg and B Points) showed the highest mean Euclidean distances between predicted and observed values, indicating lower prediction accuracy.

## Author Contributions

Conceptualisation: L.F., M.N., and P.A. Data curation: L.F., L.C., and V.R. Methodology: L.F., L.C., and M.N. Supervision: V.G. and V.R. Writing – original draft: M.D.S. Writing – review and editing: J.A.M, M.N., L.F., and M.D.S.

## Funding

The authors have nothing to report.

## Ethics Statement

This is a retro‐prospective follow‐up study with historical controls involving no drugs or medical devices. Full compliance with the principles of ethics and good clinical practice is declared. This study was exempted from review by the Medical School Institutional Review Board of the University of Michigan (HUM00160285).

## Consent

The authors have nothing to report.

## Conflicts of Interest

The authors declare no conflicts of interest.

## Supporting information


**Figure S1:** Cephalometric points of the study. Ba: Basion; S: Sella; N: Nasion; Pt: Pterygoid point; Or: Orbitale; PNS: Posterior Nasal Spine; ANS: Anterior Nasal Spine; A: Point A; B: Point B; Pg: Pogonion; Gn: Gnathion; Me: Menton; Go: Gonion; Po: Porion; Ar: Articulare; Co: Condylion.

**Table S1:** Definition of the 16 cephalometric points.

**Table S2:** Descriptive statistical analysis at first and last observations for the 144 Class III subjects.

**Table S3:** Mean absolute error (mm) on the X‐axis for the short‐term prediction.

**Table S4:** Mean absolute error (mm) on the Y‐axis for the short‐term prediction.

**Table S5:** Mean absolute error (mm) on the X‐axis for the long‐term prediction.

**Table S6:** Absolute mean error (mm) on the Y‐axis for the long‐term prediction.

**Table S7:** Pearson correlation coefficients (r) between the Euclidean distance (predicted vs. observed values) and both the age at first observation and the duration of the follow‐up interval for landmarks Go, Me, Gn, Pg, and B.

## Data Availability

The data that support the findings of this study are available from the corresponding author upon reasonable request.
