# Supplementary material for: Machine Learning‐Based Model for Predicting Short‐ and Long‐Term Growth in Untreated Class III Malocclusion
Source: Orthod Craniofac Res. 2026 May 18;29(5):954–62. doi: 10.1111/ocr.70142 (PMC13543202; doi:10.1111/ocr.70142)
Supplement: Supplementary file 2 [file OCR-29-954-s002.docx]

|  | Cephalometric point | Definition |
| --- | --- | --- |
| 1 | Basion (Ba) | lowest midpoint of the anterior border of the foramen magnum |
| 2 | Sella (S) | central midpoint of the sella turcica |
| 3 | Nasion (N) | most anterior midpoint on the sagittal plane of the frontonasal suture |
| 4 | Pterygoid point (Pt) | bilateral highest and posterior point of the pterygomaxillary fissure, defined anteriorly by the posterior surface of the tuberosity of the maxilla and posteriorly by the pterygoid processes of the sphenoid bone |
| 5 | Orbitale (Or) | bilateral point at the lower margin of the orbit |
| 6 | Anterior Nasal Spine (ANS) | most anterior midpoint on the sagittal plane of the anterior nasal spine |
| 7 | Posterior Nasal Spine (PNS) | most posterior midpoint of the bony palate |
| 8 | Point A (A) | most retruded midpoint of the anterior concavity of the maxilla between the anterior nasal spine and the alveolar process |
| 9 | Point B (B) | most retruded midpoint of the concavity of the anterior region of the mandible, between the alveolar process and the anterior symphyseal prominence |
| 10 | Pogonion (Pg) | most prominent midpoint on the sagittal plane of the mental symphysis |
| 11 | Gnathion (Gn) | midpoint of the geometric construction obtained by projecting into the anterior edge of the symphysis and the bisector of the angle formed by the facial plane (N-Me) and the mandibular plane |
| 12 | Menton (Me) | point on the most inferior border of the symphysis |
| 13 | Gonion (Go) | bilateral point of the geometric construction obtained by projecting into the mandibular angle the bisector of the angle formed by two lines: one passing from Menton (Me) to the lowest point of the posteroinferior margin of the mandible and the other from Articulare (Ar) to the highest point protruding of the posterior margin of the ramus of the mandible |
| 14 | Articulare (Ar) | bilateral point of intersection between the posterior border of the ramus and the inferior border of the posterior cranial base (occipital bone) |
| 15 | Condylion (Co) | bilateral superior point of the head of the condyle |
| 16 | Porion (Po) | bilateral point highest of the superior margin of the external auditory canal |

**Supplementary Table 1** – Definition of the 16 cephalometric points.
