# Supplementary material for: Machine Learning‐Based Model for Predicting Short‐ and Long‐Term Growth in Untreated Class III Malocclusion
Source: Orthod Craniofac Res. 2026 May 18;29(5):954–62. doi: 10.1111/ocr.70142 (PMC13543202; doi:10.1111/ocr.70142)
Supplement: Supplementary file 3 [file OCR-29-954-s003.docx]

**Supplementary Table 2** – Descriptive statistical analysis at first and last observations
for the 144 Class III subjects.

| **First observation** | | | | | |
| --- | --- | --- | --- | --- | --- |
|  | **Mean** | **SD** | **Median** | **Min** | **Max** |
| **Age** | 10.0 | 3.7 | 9.8 | 2.9 | 19.7 |
| **SNA** | 79.6 | 3.9 | 79.7 | 68.9 | 89.5 |
| **SNB** | 79.9 | 4.1 | 79.5 | 69.1 | 88.9 |
| **ANB** | -0.3 | 2.2 | 0.0 | -8.0 | 6.2 |
| **Wits** | -4.9 | 2.7 | -4.7 | -13.8 | 0.4 |
| **Co-Gn** | 104.3 | 10.8 | 104.6 | 83.1 | 135.3 |
| **SN-PP** | 8.7 | 3.6 | 8.3 | -0.5 | 18.6 |
| **SN-MP** | 35.9 | 6.0 | 36.9 | 21.4 | 52.3 |
| **Last Observation** | | | | | |
|  | **Mean** | **SD** | **Median** | **Min** | **Max** |
| **Age** | 13.8 | 3.8 | 14.0 | 5.7 | 21.7 |
| **SNA** | 79.7 | 4.1 | 79.9 | 66.7 | 88.5 |
| **SNB** | 81.3 | 4.3 | 80.8 | 70.1 | 90.2 |
| **ANB** | -1.6 | 2.5 | -1.4 | -9.1 | 3.7 |
| **Wits** | -6.1 | 3.4 | -5.7 | -16.4 | 3.0 |
| **Co-Gn** | 112.7 | 10.2 | 113.4 | 90.0 | 140.4 |
| **SN-PP** | 8.8 | 3.6 | 8.7 | -1.7 | 18.9 |
| **SN-MP** | 34.9 | 6.5 | 35.5 | 20.5 | 51.1 |

SD: Standard Deviation; Min: Minimum; Max: Maximum.
