# Supplementary material for: Machine Learning‐Based Model for Predicting Short‐ and Long‐Term Growth in Untreated Class III Malocclusion
Source: Orthod Craniofac Res. 2026 May 18;29(5):954–62. doi: 10.1111/ocr.70142 (PMC13543202; doi:10.1111/ocr.70142)
Supplement: Supplementary file 5 [file OCR-29-954-s005.docx]

**Supplementary Table 5** – Mean absolute error (mm) on the X-axis for the long-term prediction.

|  | **Mean** | **SD** | **95% CI Lower** | **95% CI Upper** |
| --- | --- | --- | --- | --- |
| **CoX** | 0.9 | 0.7 | 0.5 | 1.3 |
| **ArX** | 1.1 | 0.8 | 0.6 | 1.6 |
| **GoX** | 1.5 | 1.1 | 0.8 | 2.1 |
| **MeX** | 3.4 | 2.1 | 2.2 | 4.7 |
| **GnX** | 3.2 | 2.1 | 1.9 | 4.5 |
| **PgX** | 3.2 | 2.3 | 1.8 | 4.6 |
| **BX** | 2.3 | 1.8 | 1.2 | 3.4 |
| **AX** | 1.2 | 1.2 | 0.5 | 2.0 |
| **ANSX** | 1.6 | 1.2 | 0.8 | 2.3 |
| **PNSX** | 0.8 | 0.6 | 0.4 | 1.2 |
| **NX** | 0.8 | 0.7 | 0.4 | 1.3 |
| **SX** | 0.3 | 0.2 | 0.1 | 0.4 |
| **PoX** | 1.3 | 0.7 | 0.9 | 1.7 |
| **OrX** | 1.4 | 1.0 | 0.8 | 2.0 |
| **BaX** | 0.9 | 0.8 | 0.4 | 1.4 |
| **PtX** | 1.0 | 0.5 | 0.7 | 1.4 |

SD: Standard Deviation; CI: Confidence Interval
