# Supplementary material for: Machine Learning‐Based Model for Predicting Short‐ and Long‐Term Growth in Untreated Class III Malocclusion
Source: Orthod Craniofac Res. 2026 May 18;29(5):954–62. doi: 10.1111/ocr.70142 (PMC13543202; doi:10.1111/ocr.70142)
Supplement: Supplementary file 6 [file OCR-29-954-s006.docx]

**Supplementary Table 6** – Absolute mean error (mm) on the Y-axis for the long-term prediction.

|  | **Mean** | **SD** | **95% CI Lower** | **95% CI Upper** |
| --- | --- | --- | --- | --- |
| **CoY** | 0.8 | 0.7 | 0.4 | 1.2 |
| **ArY** | 0.8 | 0.7 | 0.3 | 1.2 |
| **GoY** | 2.4 | 1.8 | 1.3 | 3.5 |
| **MeY** | 2.1 | 2.2 | 0.8 | 3.4 |
| **GnY** | 2.1 | 2.2 | 0.8 | 3.5 |
| **PgY** | 2.8 | 2.2 | 1.5 | 4.1 |
| **BY** | 3.1 | 2.8 | 1.5 | 4.8 |
| **AY** | 2.1 | 1.2 | 1.4 | 2.8 |
| **ANSY** | 1.7 | 1.4 | 0.8 | 2.6 |
| **PNSY** | 0.9 | 0.7 | 0.5 | 1.3 |
| **NY** | 0.5 | 0.3 | 0.3 | 0.6 |
| **SY** | 0.9 | 0.8 | 0.4 | 1.4 |
| **PoY** | 0.6 | 0.5 | 0.3 | 0.9 |
| **OrY** | 0.6 | 0.6 | 0.2 | 0.9 |
| **BaY** | 0.8 | 0.6 | 0.5 | 1.2 |
| **PtY** | 0.6 | 0.4 | 0.4 | 0.8 |

SD: Standard Deviation; CI: Confidence Interval
