# Supplementary material for: Machine Learning‐Based Model for Predicting Short‐ and Long‐Term Growth in Untreated Class III Malocclusion
Source: Orthod Craniofac Res. 2026 May 18;29(5):954–62. doi: 10.1111/ocr.70142 (PMC13543202; doi:10.1111/ocr.70142)
Supplement: Supplementary file 7 [file OCR-29-954-s008.docx]

**Supplementary Table 7**. Pearson correlation coefficients (*r*) between the Euclidean distance (predicted vs. observed values) and both the age at first observation and the duration of the follow-up interval for landmarks Go, Me, Gn, Pg, and B.

| **Landmark**  **Euclidean distance** | **Age at first observation**  ***r* (P-value)** | **Time interval**  ***r* (P-value)** |
| --- | --- | --- |
| **Go** | -0.11 (0.715) | -0.07 (0.816) |
| **Me** | -0.34 (0.250) | 0.29 (0.338) |
| **Gn** | -0.30 (0.322) | 0.21 (0.499) |
| **Pg** | -0.43 (0.142) | 0.28 (0.353) |
| **B** | -0.14 (0.647) | 0.29 (0.329) |
